# Flexible Bronchoscopy for Pulmonary Bleeding and Endobronchial Blood Clot Obstruction in Patients on Extra-Corporeal Membrane Oxygenation: A Case Series †

**DOI:** 10.3390/jcm15176759

**Published:** 2026-08-31

**Authors:** Lisa Maria Valiyaveettil, Carolin Steinack, Malcolm Kohler, Silvia Ulrich, Sascha David, Thomas Gaisl

**Affiliations:** 1 Department of Pulmonology, University Hospital Zurich, 8091 Zurich, Switzerland; 2 Faculty of Medicine, University of Zurich, 8032 Zurich, Switzerland; 3 Institute of Intensive Care Medicine, University Hospital Zurich, 8091 Zurich, Switzerland

**Keywords:** bronchoscopy, pulmonary bleeding, ECMO, endobronchial blocker

## Abstract

**Background**: Extracorporeal membrane oxygenation (ECMO) is a life-saving organ support for severe circulatory or respiratory failure, but it is frequently complicated by bleeding. Pulmonary haemorrhage and consecutive airway obstruction are major contributors to morbidity and mortality. Flexible bronchoscopy (FB) is indispensable for diagnosing and managing pulmonary bleeding; however, its safety and efficacy in ECMO patients remain insufficiently studied. This analysis evaluated the feasibility, safety, and short-term physiological effects of FB in critically ill adults supported with ECMO. **Methods**: In this single-centre retrospective study, all adult ECMO patients who underwent FB for endobronchial bleeding or obstructive clot formation between January 2020 and December 2024 at the University Hospital Zurich were included. The procedural indications, diagnostic findings, haemostatic interventions, anticoagulation management, and oxygenation parameters before and after FB were analysed. The bleeding severity was graded according to the level of intervention required for haemostasis. **Results**: Sixteen patients (median age: 51.5 years, 69% male) underwent 34 FB procedures while on ECMO (69% veno-arterial). Most procedures were therapeutic (71%) and performed for haemorrhage (35%) or airway obstruction (21%). Clot removal was attempted in 85% of bronchoscopies, achieving partial or complete clearance in most cases. The median PaO_2_/FiO_2_ ratio improved from 142.8 mmHg to 161.4 mmHg (post/pre ratio: 1.36); requiring a lower FiO2 (*p* = 0.049). Of the procedures performed for active bleeding, 24% were classified as severe, and bronchial blockers were required in 26%. Bleeding control failed in 12% of cases. The ICU mortality was 62.5%, with pulmonary haemorrhage directly causing death in 25%. **Conclusions**: FB is feasible in ECMO patients and provides a measurable short-term improvement in oxygenation. However, the high mortality underscores the severity of the underlying disease. Repeat FB likely reflects the disease burden rather than procedural inefficacy.

## 1. Introduction

Extracorporeal membrane oxygenation (ECMO) is being increasingly used as a life-saving organ support for severe circulatory or respiratory failure when conventional treatments fail. Despite its clinical value, ECMO carries a high risk of complications (most notably, bleeding and thrombosis), which contribute substantially to the morbidity and mortality [1,2,3,4,5].

The pathophysiology of bleeding during ECMO is complex and multifactorial, driven by systemic anticoagulation, circuit-related contact activation, haemotrauma, hyperfibrinolysis and the underlying critical illness of the patient. The risk of spontaneous pulmonary haemorrhage increases with the duration of ECMO support and can critically impair gas exchange, often necessitating bronchoscopic intervention [2,6,7,8,9].

Flexible bronchoscopy (FB) is a minimally invasive bedside procedure routinely and widely used in the ICU for the diagnosis and treatment of lower airway pathologies. FB allows for the identification and localisation of bleeding sources and the achievement of immediate airway management with relatively low complication rates, making it an important tool in critically ill patients. It is routinely performed in patients receiving ECMO and is not itself a contradiction. Therapeutic FB is usually indicated to respond to a risk situation, generally in the context of a life-threatening haemoptysis [10,11,12,13,14]. However, evidence specific to therapeutic FB for pulmonary haemorrhage and endobronchial clot obstruction during ECMO remains limited and is largely based on case reports. Although FB is generally considered a low-risk procedure, the occurrence of acute massive haemoptysis may be fatal, particularly in patients on mechanical ventilation. Additionally, FB may entail a greater risk of complications in ECMO patients due to the use of systemic anticoagulation and a greater degree of illness in these patients. These patients are often critically ill and frequently undergo urgent interventions, requiring the discontinuation of anticoagulant or antiplatelet therapy, which can further increase the risk of thrombosis; however, continuing these agents can lead to an increased risk of haemorrhage [8,15,16,17,18].

While FB is essential for diagnosing and controlling lower airway bleeding, its therapeutic use requires balance. Although FB allows for the removal of obstructing clots and local haemostasis, repeated manipulation can provoke rebleeding and increase the risk of airway injury. A systematic evaluation is therefore needed to define the threshold between effective bleeding control and procedure-related harm [18].

This analysis aimed to evaluate the feasibility and clinical outcome of FB for pulmonary bleeding and obstruction in critically ill adult (≥18 years of age) patients undergoing ECMO.

## 2. Methods

### 2.1. Study Design and Participants

This single-centre, retrospective case series was conducted at the Department of Pulmonology at the University Hospital of Zurich. The local ethics committee reviewed the analysis (Req-2024-01552). The study was conducted in accordance with the Declaration of Helsinki. Whenever applicable, the patients (or legal representatives) provided general consent for the further use of their clinical data for research purposes [19].

Potentially eligible cases were identified through a structured search of bronchoscopy reports for documented endobronchial bleeding and/or blood-clot formation between 1 January 2020 and 31 December 2024. This search was used for case ascertainment and was not intended to establish the total number of bronchoscopies performed during the study period. The patient- and procedure-level eligibility was subsequently verified by a manual review of the electronic medical record. Those eligible were adult patients (≥18 years of age) who underwent FB between 1 January 2020 and 31 December 2024, specifically indicated for suspected endobronchial bleeding or blood-clot-related airway obstruction in the tracheobronchial tree. Endobronchial bleeding was diagnosed bronchoscopically by direct visualization of active bleeding or the presence of a blood clot within the lumen. Patients not receiving veno-venous (VV) or veno-arterial (VA) ECMO support at the time of the FB were excluded from the analysis.

### 2.2. ECMO Procedure

ECMO was initiated for patients with severe circulatory or respiratory failure with a high mortality risk despite optimal conventional therapy. All procedures followed the standard protocol of the Institute for Intensive Medicine (IFI, version 5.4) [20], and were performed under the supervision of the ECMO team. The ECMO cannulation strategy was based on the following underlying pathology: veno-venous [V-V] ECMO for severe respiratory failure and veno-arterial [V-A] ECMO for circulatory failure or cardiac arrest [21]. Cannulation was performed by a cardiac surgeon. The standard configurations were V. fem.–V. jug. for V-V ECMO and V. fem.–A. fem. for V-A ECMO. Positioning was confirmed by ultrasound or transoesophageal echocardiography (TEE). Prior to cannulation, the patients received an intra-venous heparin bolus (50–100/kg body weight) unless contraindicated. Management included continuous monitoring, daily laboratory checks, and standardized protocols for oxygenation, anticoagulation, and weaning. For analysis, anti-FXa between 0.3 and 0.7 IU/mL was considered therapeutic, and 0.2–0.3 IU/mL was considered subtherapeutic. Decannulation was performed once organ function had sufficiently recovered.

### 2.3. Bronchoscopy Procedure

All bronchoscopies were performed according to the institutional SOP of the Department of Pulmonology [22]. The procedures were conducted for diagnostic or therapeutic purposes by experienced interventional pulmonologists. The patients were preoxygenated with 100% FiO_2_ and the ventilator was set to a biphasic mode during the procedure. Continuous monitoring and an arterial blood gas analysis were part of the post-procedural evaluation.

The FB haemostatic measures for active bleeding or a high bleeding risk included local and intra-venous pharmacological management and, if required, the placement of a bronchial blocker (Cook Arndt Endobronchial Blocker Set, Bloomington, IN, USA). Local haemostatic management included topical vasoconstrictors (xylometazoline hydrochloride, 2 mL of 0.1%) and an antifibrinolytic agent (tranexamic acid, 500 mg), applied at the bronchoscopist’s discretion. Intra-venous tranexamic acid (500–1000 mg) was administered when required. The standard methods for removing clots included suction, mobilization with saline, mobilization with forceps (single-use alligator cup biopsy forceps, Olympus), and cryoprobe-assisted removal (1.1, 1.7 and 2.4 mm cryoprobe, Erbe Elektromedizin, Tübingen, Germany). The chosen method(s) were documented for each procedure [23,24].

### 2.4. Outcomes

The technical outcomes, such as the therapeutic outcomes and procedural complications, were assessed separately. Therapeutic failure or incomplete treatment was defined as failure to achieve the intended bronchoscopic treatment goal, including unsuccessful clot mobilisation or removal, a residual clot left in situ, or failure to achieve haemostasis. Premature termination because ongoing bleeding precluded safe continuation was classified as an incomplete therapeutic procedure unless the bleeding was documented as having been induced or aggravated by FB. Procedural complications were defined as new or worsened events arising during or immediately after FB and were considered attributable to the procedure.

### 2.5. Bleeding Severity Classification

The bleeding severity was categorised based on the level of the intervention required for haemostasis, as volume-based classification was unreliable in the ICU context [25]. Patients were considered to have no active bleeding when neither an active haemorrhage was observed during the procedure nor the risk of bleeding was present. The bleeding risk without an active haemorrhage was defined as fragile or hyperaemic mucosa as well as if pharmacological treatment was administered preventively. Mild bleeding referred to visible active bleeding that could be controlled by the intrabronchial instillation of pharmacological agents such as vasoconstrictors (xylometazoline) or antifibrinolytics (tranexamic acid). Severe bleeding was defined as a visible active haemorrhage requiring advanced measures, including the placement of a bronchial blocker or balloon catheter.

### 2.6. Data Collection

Relevant data were extracted from the institutional electronic medical record (KISIM-TM; Cistec^®^, Zürich, Switzerland) and entered in a structured spreadsheet for analysis. The baseline demographics and relevant clinical data during the hospitalization and ICU stay were recorded. The ECMO-related parameters included the mode, indication, cannulation site, ECMO runtime, and daily ECMO settings, including the blood flow, sweep gas flow, and oxygenator FiO_2_.

The FB-related variables included the number and timing of procedures, the indication and intent (diagnostic vs. therapeutic), the bronchoscopic findings and their regional severity, and therapeutic interventions. The overall regional severity was descriptively graded as none, mild, moderate, or severe according to the extent of the documented bronchoscopic findings. The oxygenation parameters (PaO_2_, FiO_2_, P/F ratio, PaCO_2_ and pH) were collected within a 4 h window before and after each FB. The short-term effects on gas exchange were assessed using post-/pre-procedure ratios. Anticoagulation management was documented, including daily doses of unfractionated heparin (UFH), or alternatively, a direct thrombin inhibitor (argatroban or bivalirudin) [26], as well as laboratory monitoring by anti-factor Xa activity.

### 2.7. Statistical Analysis

Descriptive statistics were used to summarize the data. Continuous variables were expressed as the mean ± standard deviation (SD), or the median with the interquartile range (IQR), as appropriate. Categorical variables were described as counts (*n*) and percentages. Changes in paired continuous variables before and after FB were analysed using a Wilcoxon signed-rank test. A *p*-value < 0.05 was considered statistically significant. The analyses were conducted using STATA v18 (STATA Corp).

## 3. Results

### 3.1. Patient Selection and Characteristics

**Figure 1:**
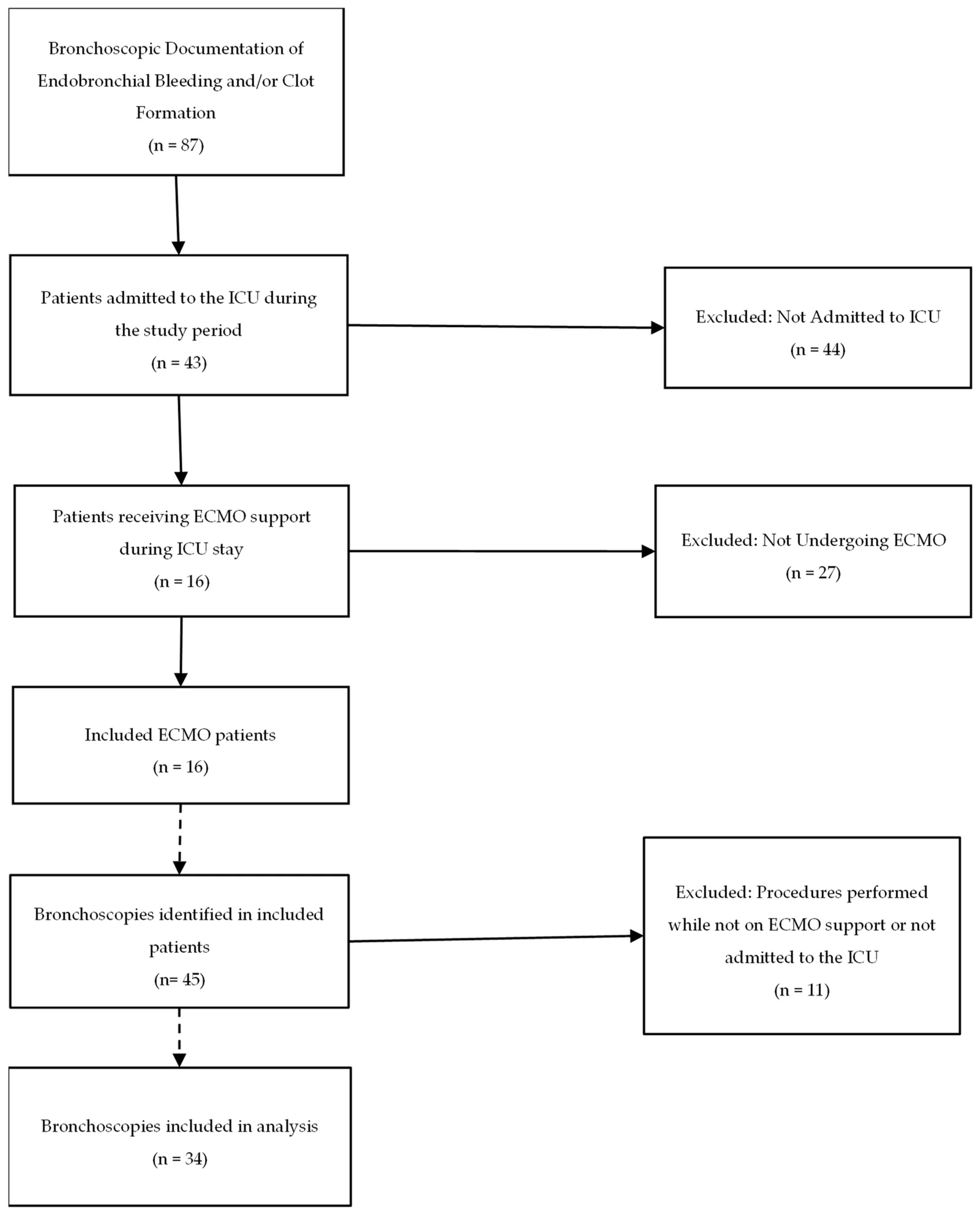
Flow diagram of patient- and procedure-level selection for the study.

**Figure 2:**
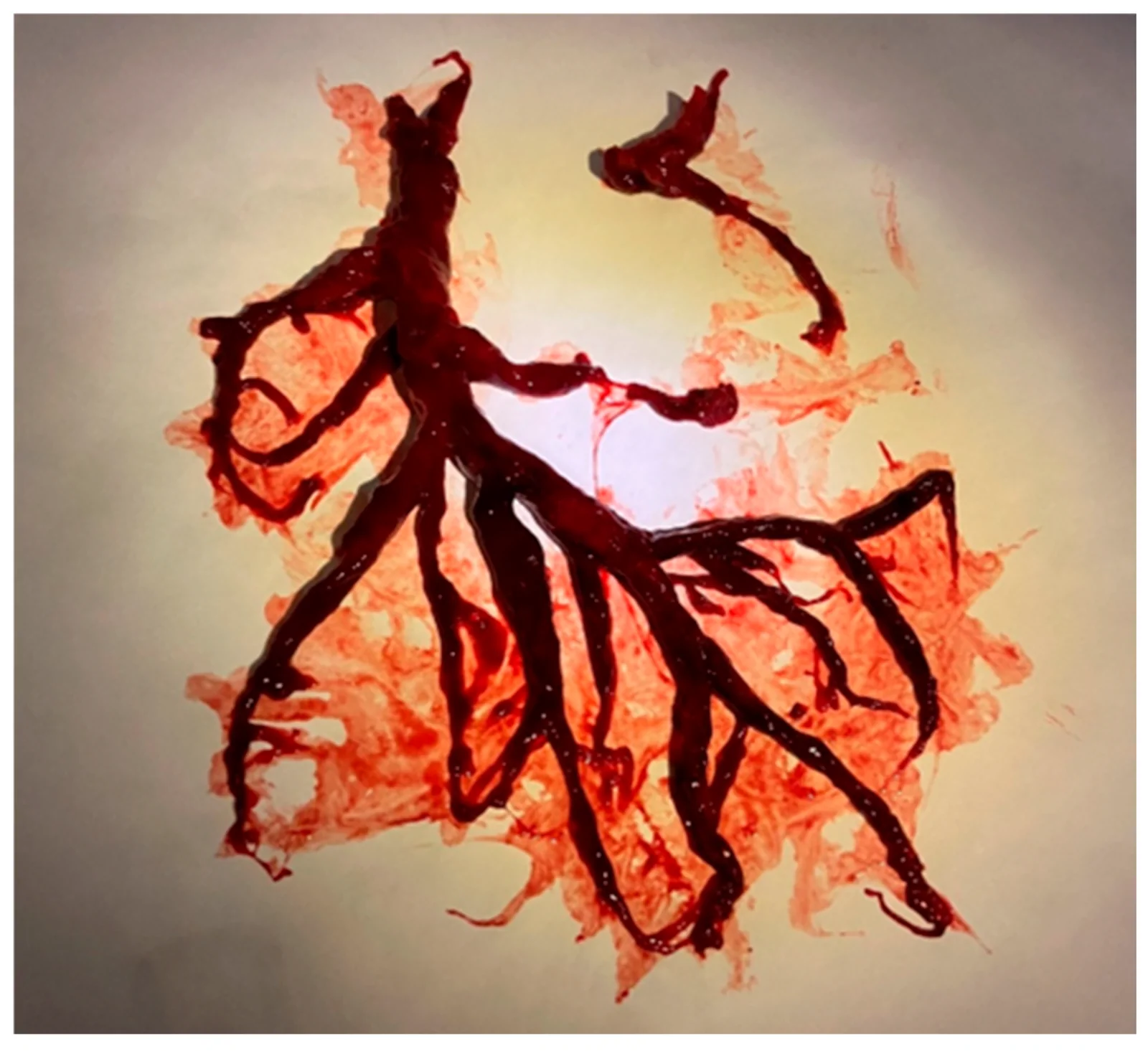
Large endobronchial blood clot removed by flexible bronchoscopy in a patient receiving extracorporeal membrane oxygenation (ECMO). The cast-like clot reproduced the segmental and subsegmental bronchial anatomy and was successfully extracted en bloc using flexible bronchoscopy, resulting in the immediate restoration of airway patency.

We reviewed 87 adult patients with FB evidence of endobronchial bleeding or clot formation (shown in Figure 1 and Figure 2). Patients were eligible if they were admitted to the ICU and received ECMO support during the study period. Ultimately, our cohort included 16 critically ill patients who met these criteria.

**Figure 3:**
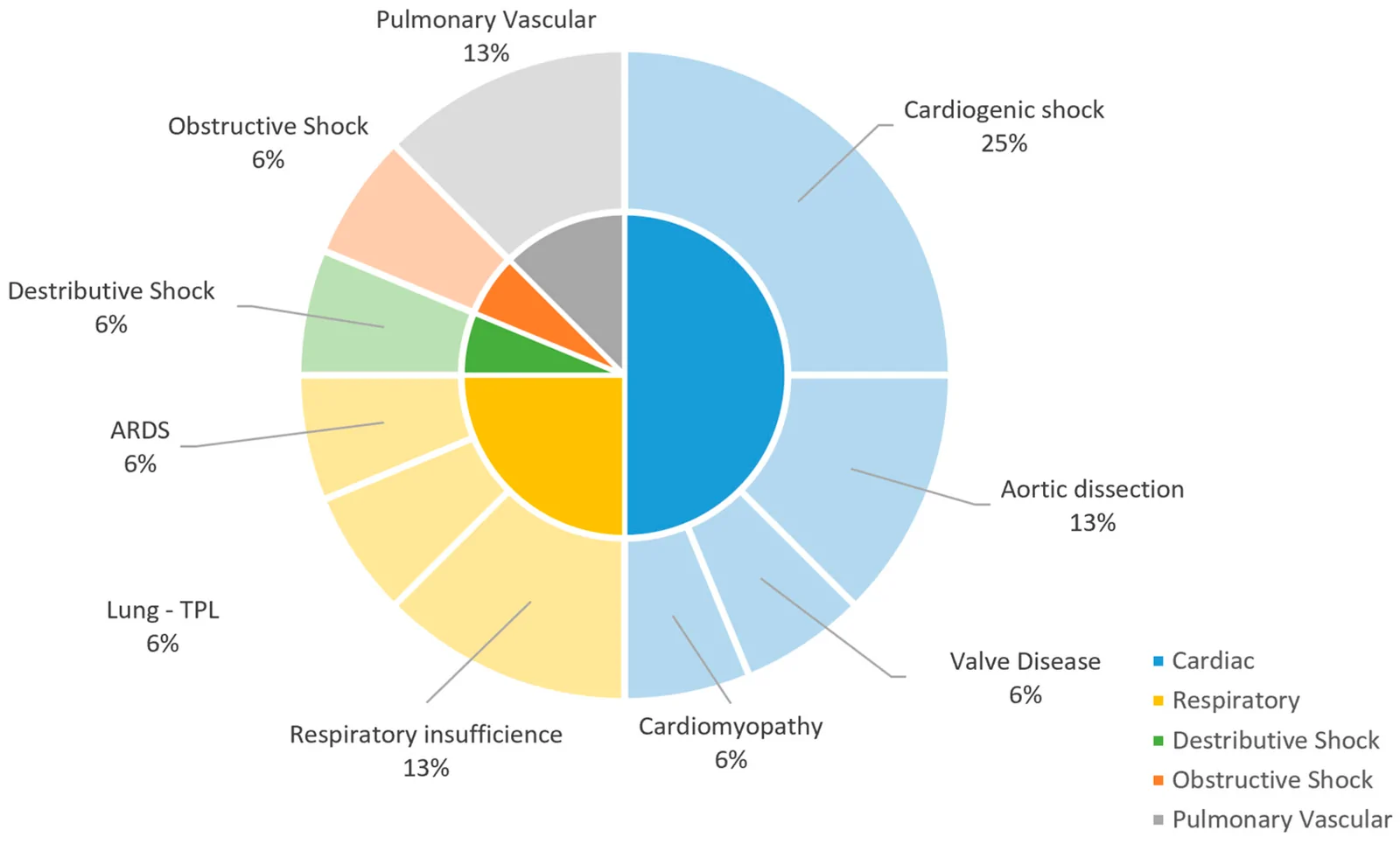
Reasons for hospitalization. Distribution of indications for hospitalization, categorized into cardiac, respiratory, distributive shock, obstructive shock, and pulmonary vascular causes. Cardiac conditions were the most common reason for admission (50%), followed by respiratory (25%) and pulmonary vascular disorders (13%). Distributive and obstructive shock each accounted for 6% of hospitalizations. Abbreviations: ARDS, acute respiratory distress syndrome; Lung-TPL, lung transplantation.

The baseline characteristics are summarized in Table 1. The median age was 51.5 years (IQR: 41–63.25) with a predominance of males (69%). The median duration of the ICU stay was 28 (IQR: 17–55) days. A cardiac diagnosis (n = 8, 50%) was the most common reason for hospitalization (shown in Figure 3). Overall, 10 of 16 patients (62.5%) died during the ICU stay. The leading causes of death were bleeding-related in six patients (37.5%), of which four cases (25%) were directly attributable to uncontrolled pulmonary haemorrhage, underlining the clinical relevance of bleeding complications in this cohort.

### 3.2. ECMO Characteristics

ECMO was initiated in all patients, with a median duration of 12 days (IQR: 8–16). FB was performed after a median of 5 days on ECMO (IQR: 1.75–12.5). Cardiac indication predominated (n = 12, 75%), and veno-arterial ECMO (n = 11, 69%) was the most frequent mode. Four patients (25%) required more than one ECMO mode during their ICU stay. The initial activated clotting time (ACT) was 208 s (IQR: 180.5–211) and the median initial heparin dose was 5000 IU (IQR: 2250–5000). The daily ECMO settings remained stable overall across intervention days, with a median blood flow of 4 L/min (IQR: 3.55–4.16) and a sweep gas flow of 3.5 L/min (IQR: 2–4.5). The median platelet count at the time of the bronchoscopy was 93 ×10^9^/L (IQR: 67.5–140.75).

### 3.3. Bronchoscopy Characteristics

#### 3.3.1. Procedures and Indications

A total of 45 FBs were performed during the study period. Eleven were excluded: in 10 cases, the patients were not on ECMO support at the time of the FB, and in one case, the patient was no longer admitted to the ICU. The final analysis therefore comprised 34 FBs. The median number per patient was one (IQR: 1–2). General FB information is shown in Table 2. Most were therapeutic (n = 24, 71%). The main indications were haemorrhage (n = 23, 35%), post-interventional follow-ups and complications (n = 10, 29%), and airway obstruction (n = 7, 21%). The most frequent access route was via an endotracheal tube (n = 23, 68%).

Nine patients underwent a single FB, whereas seven underwent multiple procedures. The ICU mortality was 5/9 (56%) in the single-FB group and 5/7 (71%) in the multiple-FB group. Patients undergoing multiple FBs also had longer ECMO support, with a median duration of 13 days (IQR: 10–24) versus 11.5 days (IQR: 3.75–17), and longer ICU stays of 29 days (IQR: 19–64.5) versus 18 days (IQR: 12.5–64.5). Given the small groups, these differences were interpreted descriptively.

#### 3.3.2. Findings

The bronchoscopic findings are summarized in Table 2, Table 3 and Table 4. The clot burden was common (n = 29, 85%), while an active haemorrhage at the time of the FB was less frequent (n = 10, 29%). In three procedures (8.8%), the findings were unclear, as no active bleeding was observed, but a suspicion of localized bleeding persisted.

Clotting primarily involved both bronchial trees, and less often the upper respiratory tract. In contrast, haemorrhage findings were less frequent overall, occurring most often in the right bronchial tree. Thrombosis was rare, and findings of secretions or mucosal irritation appeared sporadically. Pathological changes across the entire tracheobronchial tree were described in nine procedures, mostly hyperaemic mucosa or mucosal irritation. Among these, four (12%) showed extensive clotting throughout the bronchial system, and two (6%) had active bleeding.

In the upper respiratory tract, the regional severity was generally none or mild (n = 19), whereas the bronchial trees showed more moderate-to-severe involvement (n = 30; right = 17, left = 13). Across all sites, the most affected locations were the trachea (n = 25), right lower lobe (n = 19), and left upper lobe (n = 16). Clotting was the most frequent in the right lower lobe (n = 13), followed by the left lower lobe (n = 7) and left main bronchus (n = 7). Active bleeding was observed most frequently in the right main bronchus (n = 4), and the left lower lobe (n = 4), with further cases in the right middle (n = 3) and lower lobe (n = 3).

#### 3.3.3. Technical and Therapeutic Outcome

An overview of clotting and bleeding management is provided in Table 2. Clot removal was attempted in 85% of bronchoscopies, most often by suction (n = 22, 65%). In three procedures (9%), suctioning was facilitated by saline irrigation. Forceps and cryoprobes were used in more complex procedures. Mobilization failed in nine cases (26%), four procedures (12%) were aborted due to ongoing bleeding, and in three cases (9%), a residual clot was left in situ due to the location or bleeding during previous removal attempts. For haemostasis, xylometazoline hydrochloride was used in 11 procedures (32%) and tranexamic acid in eight (24%). In nine procedures (26%), the placement of a bronchial blocker was required due to the intensity of the haemorrhage, and in one procedure (3%), a balloon catheter was used. The complete cessation of bleeding was not achieved in four procedures (12%).

Documented procedure-related complications included an episode (3%) of haemodynamic instability and one episode (3%) of acute ventilatory compromise during clot extraction; these were documented as periprocedural adverse events. No pneumothorax, barotrauma, airway injury, acute oxygen desaturation, or deaths attributable to FB were documented.

#### 3.3.4. Bronchoscopy Outcome

An overview of the oxygenation and anticoagulation parameters before and after FB are presented in Table 5. The median pre-procedural FiO2 was 0.90 (IQR: 0.70–1.00), which slightly decreased post-procedure to 0.73 (IQR: 0.53–0.99; *p* for difference = 0.049). The pre-FB PaO2 values showed a median of 100.51 mmHg (IQR: 82.66–164.41), increasing to 121.51 mmHg (IQR: 89.48–141.01) post-intervention (*p* for difference: 0.135). Accordingly, the Horovitz improved, from a median of 142.75 mmHg (IQR: 98.63–207.54) before the procedure to 161.41 mmHg (IQR: 128.60–244.50) afterward (*p* for difference: 0.091). The median post/pre-Horovitz ratio was 1.36 (IQR: 0.94–2.12), suggesting a trend toward improved oxygenation following the bronchoscopic intervention. The pre- and post-procedural PaCO_2_ values suggested preserved CO_2_ elimination, with a median of 40.95 mmHg (IQR: 36.30–43.57) before and 39.60 mmHg (IQR: 35.25–43.12) after the bronchoscopy. The arterial pH remained stable, with median values of 7.40 (IQR: 7.36–7.43) pre-procedure and 7.39 (IQR: 7.36–7.43) post-procedure.

Patients undergoing a single FB (n = 9) exhibited higher baseline oxygenation levels, with a median pre-procedural Horovitz index of 205.71 mmHg (IQR: 168.00–267.00), compared to those requiring multiple procedures (n = 7), whose median was 101.67 mmHg (IQR: 95.92–113.33). The difference persisted post-procedure, with respective Horovitz indices of 243.00 mmHg (IQR: 156.00–324.75) versus 146.72 mmHg (IQR: 116.95–161.41). In contrast, PaCO_2_ remained comparable between the two groups pre- and post-treatment (single FB: 42.45 versus 37.50 mmHg; repeated FB: 39.98 versus 39.60 mmHg), with only minimal changes in the arterial pH (7.42 vs. 7.40 and 7.36 vs. 7.39, respectively), suggesting preserved ventilation and acid–base homeostasis.

Anticoagulation data were available in 27 procedures: UFH was continued in 22 cases and temporarily halted in five. In one patient, anticoagulation was switched to bivalirudin due to heparin-induced thrombotic thrombocytopenia (HITT). Anti-FXa values were available in 31 procedures, of which 10 were within the therapeutic range and 21 were within the prophylactic range. The median pre-procedural Horovitz indices were 95.83 mmHg (IQR: 58.84–112.08) and 116 mmHg (IQR: 87.97–125.53), respectively, and improved post-procedurally to 148.65 mmHg (66.66–235.55) and 127.80 mmHg (IQR: 93–168.75). The corresponding post-/pre-Horovitz ratios were 1.43 and 1.95, indicating a greater relative improvement in oxygenation among patients in the prophylactic range. When heparin was paused, the pre-procedural Horovitz index was 143.3 (IQR: 32.10–407.50), increasing to 184.5 (140.625–207.1875) post-procedure, with a post/pre ratio of 1.45.

When stratified according to the management-based bleeding severity classification, patients with severe bleeding (n = 8) had the lowest baseline oxygenation (median PaO_2_/FiO_2_: 49.65 mmHg; IQR: 31.28–195.58), but showed the greatest relative improvement (to 150.31 mmHg; IQR: 116.16–266.88), with a post/pre ratio of 3.06 (IQR: 1.38–4.21). The ratios in patients without bleeding, with a bleeding risk, and with mild bleeding were 1.26, 1.20, and 1.66, respectively.

At the time of the FB, 16 procedures were performed during VA-ECMO, six during VVA-ECMO, and 12 during VV-ECMO. The median PaO_2_/FiO_2_ ratio decreased from 205.71 mmHg (IQR: 111.00–250.50) to 160.00 mmHg (IQR: 108.00–243.00) during VA-ECMO, with a median individual post/pre ratio of 0.85. In contrast, it increased from 51.65 mmHg (IQR: 47.23–57.01) to 71.63 mmHg (IQR: 65.34–77.53) during VVA-ECMO and from 101.08 mmHg (IQR: 76.69–112.50) to 136.24 mmHg (IQR: 96.31–216.64) during VV-ECMO, with corresponding median individual ratios of 1.35 and 1.33. Because of the small subgroups and repeated procedures within patients, these findings were interpreted descriptively.

## 4. Discussion

Although ECMO support is lifesaving, complications are common and may lead to permanent injury or death. Bleeding is among the most frequent complications, with the lungs being a common site for major haemorrhage [14,27]. Arachchillage et al. [28] reported that massive haemorrhage associated with ECMO occurs in 30.9% of patients, and pulmonary bleeding accounts for 26%. Given the high incidence of pulmonary haemorrhage during ECMO, FB is an indispensable tool in the intensive care unit due to its important applications and significant effect on patient care. The development of several endobronchial topical treatments has revolutionized the role of FB in managing haemoptysis [29,30].

Herein, we describe our institution’s experience utilizing FB for the management of endobronchial bleeding and clotting in 16 patients supported with ECMO. Most bronchoscopies were performed with therapeutic intent and proved feasible despite systemic anticoagulation and critical illness. Clot removal was successful in many procedures, whereas achieving haemostasis was more challenging, often requiring advanced interventions such as bronchial blockers. This highlights the prognostic significance of bleeding, as it was strongly linked to mortality in several studies [27,31]. FB consistently resulted in short-term improvements in oxygenation, although the PaO_2_/FiO_2_ ratio remained low, reflecting the patients’ critical condition. A previous study [32] assessing the impact of FB on gas exchange also reported an improvement in oxygenation. Patients requiring repeated FBs had lower baseline oxygenation, while severe bleeding cases, despite the poorest initial values, showed the greatest relative improvement. This pattern likely reflects the relief of acute airway obstruction and improved alveolar ventilation once the clots were cleared, underscoring that the physiological benefit of FB is greatest when the mechanical impact of bleeding is most pronounced. Overall, the ICU mortality was very high (62.5%), with pulmonary haemorrhage causing death in 25% of the patients, underlining its prognostic significance and acting as the central focus of this study.

FB is widely regarded as safe in critically ill patients [32,33,34], including those on ECMO, but it has predominantly been reported in the context of diagnostic rather than therapeutic intervention [1,13]. There is limited evidence on using FB to treat pulmonary bleeding during ECMO, with most reports being case studies or small series [14,35,36,37]. Pitcher et al. [36] and Zhou et al. [35] described bronchoscopic techniques for massive haemorrhage, while Zhou et al. [35] also reported bronchial arteriography with embolization and temporary endotracheal tube clamping under full ECMO support as rescue strategies after conventional measures had failed. Neither of these additional strategies were used in our cohort. Despite these reports, evidence regarding therapeutic FB for endobronchial bleeding during ECMO remains limited.

FB appeared feasible in this critically ill cohort; however, its safety requires cautious interpretation. Most unfavourable procedural outcomes reflected therapeutic non-success rather than definite procedure-related complications. Two periprocedural adverse events were documented: one episode of haemodynamic instability and one of an acute ventilatory compromise. Nevertheless, the small cohort, retrospective ascertainment, absence of a control group, and severity of the underlying illness preclude definitive conclusions regarding procedural safety.

Our analysis extends the limited evidence in this field by providing a detailed assessment of airway clearance and haemostasis, specifically looking at short-term effects on gas exchange. So far, only a few studies have addressed the oxygenation dynamics in this setting. Smeijsters et al. [32] studied how FB affects gas exchange by measuring the Horovitz index at different times, finding a significant increase in the PaO_2_/FiO_2_ ratio post-FB. Our analysis is novel because it focuses on short-term oxygenation in critically ill patients on ECMO, thereby offering insight into the efficacy of therapeutic FB in this high-risk population. The oxygenation changes differed descriptively according to the ECMO configuration: the PaO_2_/FiO_2_ ratio decreased during VA-ECMO, but improved during VVA- and VV-ECMO. However, these differences cannot be attributed to the ECMO mode itself because of the small subgroups, repeated procedures, mode changes, and substantial differences in the baseline oxygenation and underlying clinical indications.

Regarding the frequency of bronchoscopic procedures, the existing data are limited. One case study [35] suggests that repeated FB might risk worsening bleeding. In contrast, our findings highlight that repeat procedures were performed to maintain airway patency. Patients undergoing multiple bronchoscopies had lower baseline oxygenation, a numerically higher ICU mortality (71% vs. 56%), and longer ECMO support and ICU stays, suggesting that repeat FB may serve as a surrogate marker for severe pulmonary compromise rather than procedural inefficacy. Clinically, the need for multiple bronchoscopies could thus serve as a red-flag indicator for a refractory pulmonary pathology or an ongoing bleeding tendency. Unlike other studies [11,35,38], our management-based bleeding severity classification, inspired by the framework proposed by Bernasconi et al. [25], focuses on the interventions required to achieve haemostasis. This approach provides an alternative to traditional volume-based definitions and may better reflect the clinical relevance and guide treatment strategies more effectively in this complex patient group. Our literature search did not yield any studies evaluating the oxygenation in relation to bleeding severity. While several studies [7,9,31,39] have reported the role of the anticoagulation intensity in bleeding in ECMO patients, our subgroup analyses of anti-FXa values did not show a clear association with oxygenation outcomes. The observed mortality rate of 62.5% was higher than that reported in large ECMO registries (54–57%) [3,4,39,40]. This may partly reflect the high proportion of patients supported with VA ECMO, as well as the occurrence of pulmonary haemorrhage during ECMO support. As Deinzer et al. [40] reported, both VA support and bleeding complications are associated with an increased mortality, and because VA patients are more prone to bleeding, the elevated risk in our cohort is plausible.

This study has several limitations. Its retrospective nature makes room for potential biases. Due to the small sample size, the findings remain to be validated by subsequent clinical trials with larger sizes. The point regarding *p*-values is well taken. The sample size was too small to allow for meaningful statistical testing, and a larger sample size would be required to draw robust conclusions. Nevertheless, the current data already allow for a tentative assessment. Another limitation is that ECMO support limits the interpretation of absolute oxygenation parameters; the ECMO settings together with preserved PaCO_2_ elimination and a stable pH suggest that major changes in extracorporeal support or ventilation were unlikely to account for the observed short-term differences in oxygenation.

In this retrospective case series, FB was feasible for managing endobronchial bleeding and clot-related airway obstruction during ECMO and was associated with favourable short-term oxygenation trends. Poor overall outcomes reflected the critical illness of the cohort, while repeated procedures appeared to represent a greater disease severity rather than procedural inefficacy. However, the small cohort and retrospective design preclude firm conclusions regarding procedural safety or efficacy. Larger prospective multicentre studies are needed to validate management-based bleeding classifications, clarify the impact of anticoagulation, and define optimal bronchoscopic strategies for this population.

## Figures and Tables

**Table 1:** Baseline patient characteristics and ECMO-related characteristics.

Included Patients *n* = 16		Data Available on *n* (%)
Age in years, median (IQR)	51.5 (41–63.25)	16 (100%)
Male sex, *n* (%)	11 (69%)	16 (100%)
Hospitalization length in days, median (IQR)	29 (17.25–77.25)	
Hospitalization reason, *n* (%)		16 (100%)
Cardiac	8 (50%)	
Respiratory	4 (25%)	
Distributive shock	1 (6%)	
Obstructive shock	1 (6%)	
Pulmonary vascular	2 (13%)	
Length of ICU stay in days, median (IQR)	28 (17–55)	16 (100%)
ICU outcome, *n* (%)		16 (100%)
Deceased	10 (62.5%)	
Survived	6 (37.5%)	
Cause of death during ICU stay		10 (62.5%)
Bleeding-related	6 (31.3%)	
Pulmonary	4 (25%)	
Other bleeding sites	2 (12.5%)	
Cardiac (non-bleeding)	2 (12.5%)	
Other neurological/multiorgan failure	2 (12.5%)	
ECMO indication, n (%)		
Cardiac	12 (75%)	
Respiratory	4 (25%)	
Mixed	3 (19%)	
Mechanical/other	2 (13%)	
Type of ECMO support, n (%)		
Veno-arterial	11 (69%)	
Veno-venous	5 (31%)	
Combined	5 (31%)	
ECMO runtime in days, median (IQR)	12 (8–16)	
Veno-arterial	14 (10.5–16)	
Veno-venous	16 (12–19)	
Combined	12 (2.5–14.5)	
Pre-bronchoscopy ECMO days	5 (1.75–12.5)	
ASA classification during initiation, n (%)		11
ASA 3	3	
ASA 4	7	
ASA 5	1	
Platelet count at bronchoscopy, ×10^9^/L, median (IQR)	93 (67.5–140.75)	34
Initial ACT in seconds, median (IQR)	208 (180.5–211)	10
Initial heparin dose, median (IQR)	5000 (2250–5000)	11
Cannulation sites, n (%)		
Infusion cannula		
Artery: femoral	9 (56.25%)	
Artery: subclavian	2 (12.5%)	
Artery: pulmonary	1 (6.25%)	
Artery: axillary	1 (6.25%)	
Vein: jugular	4 (25%)	
Vein: femoral	1 (6.25%)	
Drainage cannula		
Vein: femoral	13 (81.25%)	
Vein: jugular	3 (18.75%)	

**Table 2:** Bronchoscopic characteristics.

Included Bronchoscopies *n* = 34		Data Available on *n* (%)
Number per patient, median (IQR)	1 (1–2.25)	34 (100%)
Single bronchoscopy		
Repeated bronchoscopy		
Procedure intent, *n* (%)		34 (100%)
Therapeutic	24 (70.4%)	
Diagnostic	6 (17.6%)	
Both	4 (11.8%)	
Primary indication, *n* (%)		32 (94%)
Bleeding/haemorrhage	12 (35.3%)	
Post-interventional follow-up and complications	10 (29.4%)	
Airway obstruction	7 (20.6%)	
Infectious/inflammatory	2 (5.9%)	
Surveillance of progressive/chronic respiratory symptoms	1 (2.9%)	
Access site, *n* (%)		34 (100%)
Via endotracheal tube	23 (68%)	
Via tracheostomy	9 (26%)	
Via bite block	2 (6%)	
Bleeding incidents, *n* (%)		34 (100%)
Active bleeding		
Yes	10 (29%)	
No	21 (62%)	
Unclear	3 (9%)	
Clotting		
Yes	29 (85%)	
No	5 (15%)	
Clotting management techniques used, *n* (%)		29 (85%)
Suctioning	22 (65%)	
Saline irrigation	3 (9%)	
Forceps	4 (12%)	
Cryoprobe	4 (12%)	
Bleeding management techniques used, *n* (%)		17 (50%)
Bronchial blocker	9 (26%)	
Balloon catheter	1 (3%)	
Xylometazoline hydrochloride	11 (32%)	
Tranexamic acid	8 (24%)	
Technical and therapeutic outcomes, *n* (%)		
Airway clearance	29 (85%)	
Clearance attempted, successful	17 (50%)	
Clearance attempted, unsuccessful	9 (26%)	
Clearance not attempted	3 (9%)	
Haemostasis		
Bleeding controlled by bronchoscopic measures	6 (18%)	
Procedure aborted due to bleeding	4 (12%)	
Procedure terminated because of ongoing haemorrhage	4 (12%)	
Procedure-related adverse events		
Hemodynamic instability	1 (3%)	
Ventilatory compromise	1 (3%)	

**Table 3:** Localization of bronchoscopic findings by anatomical region.

Localization [n]	General Findings	Clotting	Bleeding
Entire bronchial tree	9	4	2
URT (N = 29)			
Pharynx	1		
Larynx	2		
Trachea	25	6	2
Main carina	6	3	1
RBT (N = 33)			
Right main bronchus	8	5	4
Right upper lobe	2	3	0
Bronchus intermedius	3	3	0
Middle lobe	7	6	3
Lower lobe	19	13	3
LBT (N = 26)			
Left main bronchus	10	7	1
Left upper lobe	16	6	2
Left lower lobe	9	7	4

URT: upper respiratory tract, RBT: right bronchial tree, LBT: left bronchial tree.

**Table 4:** Bronchoscopic findings by anatomical region.

	URT (N = 29)	RBT (N = 33)	LBT (N = 26)
Clotting, n (%)	10	20	19
Haemorrhagic findings, n (%)			
Fresh blood	2	2	
Old blood	3	6	3
Thrombosis, n (%)	2	3	1
Secretion, n (%)	2	4	3
Irritated mucosa, n (%)	3	3	6
Overall severity of bronchoscopic findings			
None	16	4	3
Mild	3	3	
Moderate	2	9	8
Severe	6	8	5
Unknown	2	9	10

**Table 5:** Oxygenation parameters before and after bronchoscopy in ECMO patients.

Analysis Group	N	Pre-Horovitz Index, mmHg Median (IQR)	Post-Horovitz Index, mmHg Median (IQR)	Post/Pre-Ratio Median (IQR)
Overall cohort (n = 16, 34 procedures)		142.75 (98.63–207.54)	161.41 (128.6–244.5)	1.36 (0.94–2.12)
By number of bronchoscopies				
1 bronchoscopy	9	205.71 (168–267)	243.00 (156–324.75)	1.27 (0.86–2.82)
>1 bronchoscopy	7	101.67 (95.92–113.33)	146.72 (116.95–161.41)	1.42 (1.17–1.91)
By anticoagulation (anti-FXa, n = 31)				
Therapeutic range (0.3–0.7)	10	95.83 (58.84–112.08)	148.65 (66.66–235.55)	1.43 (1.09–2.12)
Prophylactic range (0.2–0.3)	21	116 (87.97–125.53)	127.80 (93–168.75)	1.95 (1.33–3.23)
By bleeding severity (n = 34)				
No active bleeding	15	110.83 (74.55–205.50)	102 (70.31–196.27)	1.26 (0.79–1.41)
Bleeding risk without active bleeding	7	120.00 (86.18–186.86)	144.38 (113.91–192.56)	1.20 (0.83–1.71)
Mild bleeding	2	93.75 (84.38–103.13)	165.63 (125.94–205.31)	1.66 (1.41–1.92)
Severe bleeding	8	49.65 (31.28–195.58)	150.31 (116.16–266.88)	3.06 (1.38–4.21)
By ECMO configuration (n = 34)				
VA (veno-arterial)	16	205.71 (111.1–250.5)	160.00 (108–243)	0.85 (0.7–2.44)
VVA (veno-veno-arterial)	6	51.65 (47.23–57.01)	71.63 (65.34–77.53)	1.35 (1.27–1.45)
VV (veno-venous)	12	101.08 (76.6875–112.5)	136.24 (96.31–216.64)	1.33 (1.1–2.43)

## Data Availability

The anonymized dataset supporting this study is available from the corresponding author upon reasonable request.
